# The fitness of chemotrophs increases when their catabolic by‐products are consumed by other species

**DOI:** 10.1111/ele.13397

**Published:** 2019-10-14

**Authors:** Mayumi Seto, Yoh Iwasa

**Affiliations:** ^1^ Department of Chemistry, Biology, and Environmental Sciences Nara Women’s University Kita‐Uoya Nishimachi Nara 630‐8506 Japan; ^2^ Department of Bioscience, School of Science and Technology Kwansei Gakuin University Gakuen 2‐1, Sanda‐shi Hyogo 669‐1337 Japan

**Keywords:** Abundant Resource Premium, chemotrophs, material network, mathematical model, microbial ecology, mutualism, niche construction, niche expansion, syntrophy

## Abstract

Chemotrophic microorganisms synthesise biomass by utilising energy obtained from a set of chemical reactions that convert resources to by‐products, forming catabolic interactions. The amount of energy obtained per catabolic reaction decreases with the abundance of the by‐product named as the ‘abundant resource premium’. Consider two species, Species 1 and 2, Species 1 obtains energy from a reaction that converts resource A to by‐product B. Species 2 then utilises B as its resource, extracting energy from a reaction that converts B to C. Thus, the presence of Species 2 reduces the abundance of B, which improves the fitness of Species 1 by increasing the energy acquisition per reaction of A to B. We discuss the population dynamic implication of this effect and its importance in expanding a realised niche, boosting material flow through the ecosystem and providing mutualistic interactions among species linked by the material flow. Introducing thermodynamics into population ecology could offer us fundamental ecological insights into understanding the ecology of chemotrophic microorganisms dominating the subsurface realm.

## Graphical abstract

We developed a simple mathematical model for a microbial mutualistic interaction in which the catabolic by‐product of the first species is utilized as a catabolic resource by the second species (a non‐feeding catabolic interaction), which has been neglected in theoretical ecology. Our modeling study shows how the consumption of the by‐product of species by others reduces the minimum energy demand for persistence, expands the realized niche, and boosts material flow.

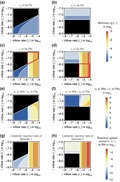

## Introduction

Trophic interactions have been considered to be the base for material flows through communities in which the anabolic product (organic matter) of a species is consumed by other species. All organisms use energy to create organic matter (anabolism). To generate energy (catabolism), phototrophs obtain energy from sunlight, meanwhile chemotrophs acquire their energy from a set of chemical reactions that convert inorganic and organic substances to by‐products. The energy‐harvesting chemical reactions of chemotrophs significantly directly and indirectly involve the flows of carbon, nitrogen, sulphur and several trace elements through the ecosystem (Konhauser 2007; Falkowski *et al.* 2008; Borch *et al.* 2010; Fenchel *et al.* 2012; Schlesinger & Bernhardt 2013). An organism generating energy in this way is functioning as a catalyst for the conversion of an element from one elemental chemical form to another chemical form (Box 1). Most animals and fungi are chemotrophs that utilise organic matter as anabolic and catabolic sources. Part of organic matter undergoes a catabolic reaction with oxygen (O_2_) and releasing carbon dioxide (CO_2_), without the carbon accumulating in the organism’s body as organic carbon pool. Some bacteria and archaea do not even require organic matter to generate energy. Iron‐oxidising bacteria obtain energy from the reaction of ferrous iron (Fe^2+^) with O_2_, releasing an iron oxide (Neubauer *et al.* 2002; Emerson *et al.* 2010). Indeed, the catabolic reactions of microbes are crucial for driving biogeochemical cycles (Falkowski *et al.* 2008).

Box 1 **Energy acquisition per reaction and the abundant resource premium**

The energy‐harvesting chemical reactions mostly involve the transfer of electrons between materials, which creates an accompanying imbalance of protons (hydrogen ions, H^+^) across cell membranes. This produces a proton motive force, which is used by cells to create ATP (Mitchell 1961, 2011). This type of fuel cell reaction, referred to as an oxidation–reduction (redox) reaction, is used by all chemotrophic organisms to synthesise ATP through the utilisation of materials available in the ecosystem.

Although the energy‐harvesting reaction often involves many different substances, the essential process is mainly the electron transfer between an electron donor substance and an electron acceptor substance; the other molecules are only required to balance the stoichiometry of the reaction. This can be summarised as follows:

(1) $$n_{1}A_{d}+n_{2}B_{a}\to n_{3}A_{a}+n_{4}B_{d},$$

where *A_d_* and *B_a_* are the reactants (resources), *A_a_* and *B_d_* are the products of the reaction (by‐products), *n_i_* are stoichiometric coefficients, and the subscripts *a* and *d* indicate electron acceptor and electron donor, respectively. *A_d_* becomes *A_a_*, the oxidised form of *A*, with the loss of one or more electrons (e.g. glucose C_6_H_12_O_6_ is converted to CO_2_ through aerobic respiration and Fe^2+^ is converted to an iron oxide through iron oxidation). *B_a_* becomes *B_d_*, the reduced form of *B*, with the gain of one or more electrons. The maximum energy acquisition per mole for such a reaction is given by −∆*G*, calculated as follows:

(2a) $$-\Delta G=-\Delta G^{o}+RT\ln\frac{{\left\{A_{d}\right\}}^{n_{1}}{\left\{B_{a}\right\}}^{n_{2}}}{{\left\{A_{a}\right\}}^{n_{3}}{\left\{B_{d}\right\}}^{n_{4}}},$$

where −Δ*G*º denotes the negative change in the standard Gibbs energy of reaction (in kJ mol^−1^), given by

(2b) $$-\Delta G^{{}^{\circ }}=n_{1}\Delta_{f}G_{A_{d}}^{{}^{\circ }}+n_{2}\Delta_{f}G_{B_{a}}^{{}^{\circ }}-\left(n_{3}\Delta_{f}G_{A_{a}}^{{}^{\circ }}+n_{4}\Delta_{f}G_{B_{d}}^{{}^{\circ }}\right),$$

where ° indicates standard state condition where all reactants and products have activity = 1. Its value depends on Δ*_f_* $G_{x}^{{}^{\circ }}$, the change in the standard Gibbs energy of formation of substance *x* (here, *x* = *A_d_*, *B_a_*, *A_a_* or *B_d_*). Δ*_f_* $G_{x}^{{}^{\circ }}$ is a constant intrinsic to substance *x* under the standard conditions. Because −Δ*G*º is the difference between the sum of *n_i_*Δ*_f_G*º for the reactants and the sum of *n_i_*Δ*_f_G*º for the by‐products, it is a reaction‐specific quantity determined by the combination of reactants and by‐products (Fig. 2a). Δ*_f_* $G_{x}^{{}^{\circ }}$ values are available from the CHNOSZ package for R (://chnosz.net). In general, Δ*_f_* $G_{x}^{{}^{\circ }}$ values for inorganic substances are summarised in standard physical chemistry books (Haynes 2013).

The second term of the right‐hand side of eqn 2a represents the abundant resource premium (ARP), where *R* is the gas constant (*R* = 8.13 × 10^−3^ kJ mol^−1^), *T* is absolute temperature and {X} represents the activity of X, calculated as {X} = γ*_x_*[X], where γ*_x_* is the activity coefficient of X and [X] is its molar concentration. Activity coefficients approach 1 (therefore, the activity of X becomes equivalent to its concentration) only when (a) the solution is extremely dilute (ionic strength is essentially 0) and (b) for neutral species under relatively low temperatures and pressures. $\frac{{\left\{A_{d}\right\}}^{n_{1}}{\left\{B_{a}\right\}}^{n_{2}}}{{\left\{A_{a}\right\}}^{n_{3}}{\left\{B_{d}\right\}}^{n_{4}}}$ is a reaction quotient, often denoted by *Q*. ARP is determined by *T* and *Q* and its contribution to −Δ*G* can be greater at higher temperatures depending on the temperature sensitivity of Δ*G*º. The contribution of higher temperatures to ARP is constrained because the range of temperatures at which organisms are able to survive is limited. In an ambient temperature from −10 ºC to 80 ºC, at which psychrophiles or thermophiles can thrive (e.g. Amend & Shock 2001; Price & Sowers, 2004), ARP is within the range 10^−10^ ≤ *Q* ≤ 10^10^, which corresponds to −66.1 kJ mol^−1^ ≤ ARP ≤ 66.1 kJ mol^−1^ (Fig. 2b). ARP has a significant effect on the energy acquisition per reaction only when −Δ*G*º is of the same order of magnitude as the ARP. The energy acquisition per reaction can be insensitive to ARP for reactions where −Δ*G*º> 1000 kJ mol^−1^, such as those involving aerobic respiration in which C_6_H_12_O_6_ reacts with O_2_. However, for some chemotrophic bacteria that harness a reaction for which −Δ*G*º is small, ARP can have a significant effect on their population dynamics, such as methanogens, iron‐oxidising bacteria and sulphate reducers (Kral *et al.* 1998; Emerson *et al.* 2010; Hoehler & Jørgensen 2013). It should be noted that the contribution of ARP to −Δ*G* also changes with change in pressure and coexisting chemical substances. Detailed explanations are found in general textbooks on physical chemistry.

**Figure 1:**
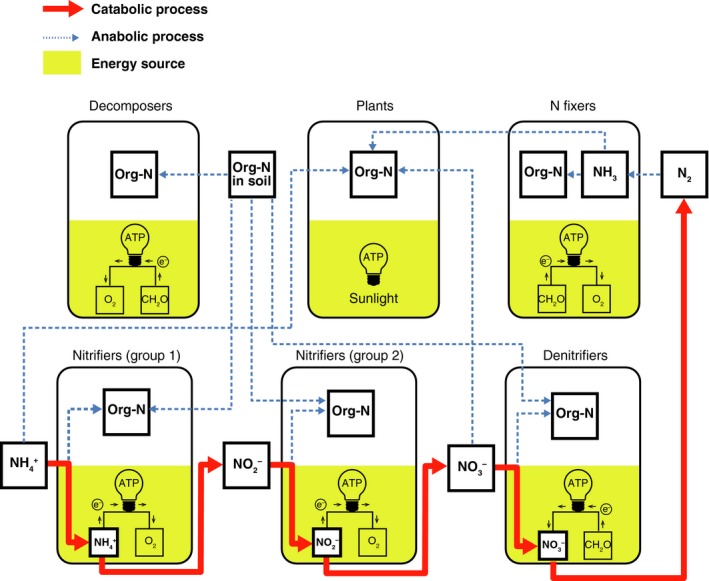
Nitrogen (N) flow, including both anabolic processes (dashed blue arrows) and catabolic processes (red arrows). The N fractions utilised as catabolic sources are transformed into other forms of N and are eventually released into the environment.

The typical diagrams representing material flow (particularly carbon and nitrogen flow) through an ecosystem often do not separately illustrate anabolic and catabolic processes. Catabolic processes are ubiquitous in nature and comprise non‐trophic flows well known in ecology such as the conversion of ammonia to dinitrogen as illustrated below. The catabolic by‐product for one microbe often acts as a catabolic resource for other microbes, forming catabolic interactions. Methanogens convert hydrogen gas and CO_2_, the catabolic by‐product of aerobic microbes, to methane to generate energy (Jones *et al.* 1985; Kral *et al.* 1998). Another well‐known example is the nitrogen cycle (Fig. 1). The nitrogen flows shown as dashed blue arrows are connected based on the anabolic processes. Nitrogen‐fixing bacteria obtain energy from aerobic respiration, synthesising ammonia (NH_3_) from nitrogen gas in the air; this boosts the pool of organic nitrogen available for other organisms. Organic nitrogen accumulates in the plant biomass through the uptake of inorganic nitrogen from soil or NH_3_ from the nitrogen symbiosis with nitrogen‐fixing bacteria (Herridge *et al.* 2008). Nitrogen translocates into the soil when plants die, is accumulated by decomposers as their organic nitrogen pool and finally forms NH_3_, or the ammonium ion (NH_4_^+^), again. Some of the nitrogen in NH_3_ may be absorbed by plants. In addition to these flows, bacteria in the genera *Nitrosomonas*, *Nitrosospira* and *Nitrosococcus* (group 1 in Fig. 1) obtain energy by utilising some of the acquired NH_3_ nitrogen in a reaction with O_2_, immediately releasing nitrite ions (${\text{NO}}_{2}^{-}$); and bacteria in the genera *Nitrobacter*, *Nitrococcus* and *Nitrospina* (group 2 in Fig. 1) then obtain energy by utilising some of this ${\text{NO}}_{2}^{-}$ in a reaction with O_2_, releasing NO_3_^−^ (Konhauser 2007). Denitrifying bacteria subsequently acquire energy from a reaction that uses NO_3_^−^ and organic matter and releases N_2_ and CO_2_. In this example, each species of bacteria catalyses only one catabolic reaction using an inorganic nitrogen substance, with several different species involved in the nitrogen cycle. The example also shows that the catalysis of material flow for energy acquisition provides an opportunity for different species to interact with each other independent of feeding.

In traditional microbial ecology, the relationship between species belonging to groups 1 and 2 in Fig. 1 is commensalism, in which the second species receives benefit from the presence of the first, which provides it with catabolic resources, but the first species receives no benefit from the presence of the second. However, the presence of the second species can be a benefit to the first species: the reduced abundance of the by‐product of the first species improves its energy acquisition per reaction.

**Figure 2:**
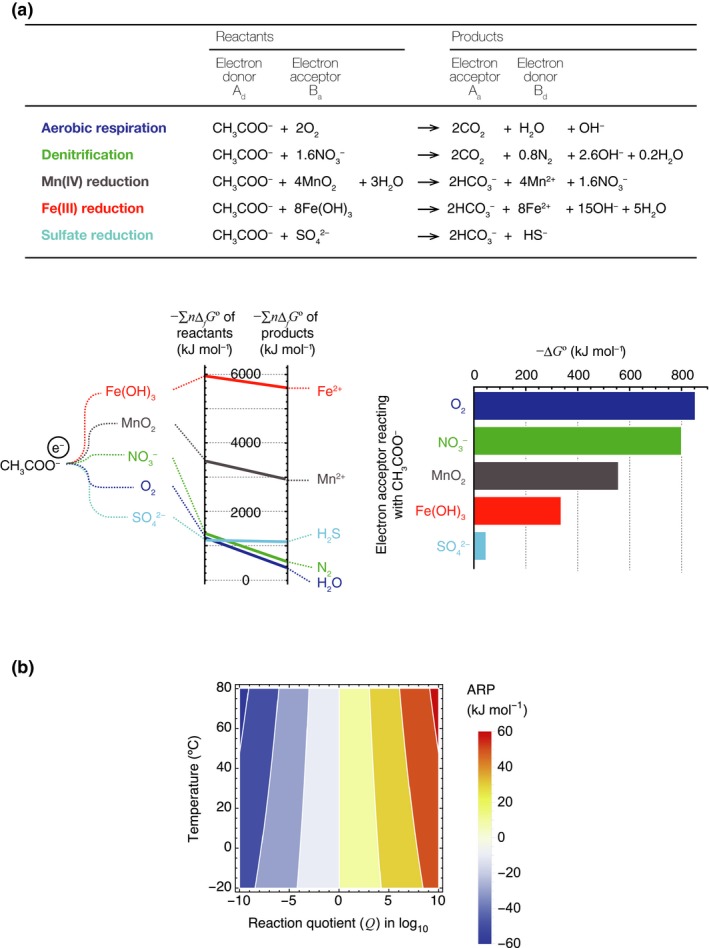
The first and second terms of the change in Gibbs energy in eqn 2a. (a) The negative change in the standard Gibbs energy, −∆*G*°, for a reaction that utilises CH_3_COO^−^ as an electron donor with various electron acceptors. These examples of reactions and the values of −∆*G*° (at 273.15 K, 1 atm and pH = 7) were adapted from Konhauser (2007). −∆*G*° corresponds to the difference between the sum of the change in the standard Gibbs energy of formation of the reactants multiplied by their stoichiometric coefficient *n_i_*Δ*_f_G*º and the same for the by‐products (see Box. 1). −∆*G*° at constant temperature and pressure is determined by the combination of reactants and by‐products. (b) How the magnitude of the abundant resource premium (ARP = *RT* ln *Q*) varies with the reaction quotient *Q* and the ambient temperature *T.*

Energy acquisition per reaction is thermodynamically constrained by the negative change in the Gibbs energy of the reaction, −∆*G* (kJ mol^−1^), which depends on the abundances of both the resources (reactants) and the by‐products of the catabolic reaction (Box 1, Fig. 2). Because chemotrophic organisms can only generate energy from a reaction with positive −∆*G* (negative ∆*G*), in the field of geochemistry, there is an emerging approach to understand microbial activities and communities as a function of −∆*G* or other thermodynamic properties (Amend & Shock 1998, 2001; Macur *et al.* 2004; Inskeep *et al.* 2005; Jin & Bethke 2005, 2007; Dale *et al.* 2006; LaRowe *et al.* 2012; Seto 2014; Nakamura & Takai 2014; LaRowe & Amend 2015; Seto *et al.* 2019); however, this approach has seldom been applied in theoretical ecology. −∆*G* increases with the abundance of catabolic resources and decreases as the by‐products become more abundant. This effect, which we named the ‘abundant resource premium’ (ARP) (Seto & Iwasa 2019), predicts the mutualism between groups 1 and 2 in Fig. 1. In reality, the ARP‐driven mutualism in a mixed microbial culture was first confirmed for a culture of *Methanobacillus omelianskii* (Baker 1939) and has been widely recognised in microbiology, especially in close association with the field of biogeochemistry (Hoehler *et al.* 1994; Boetius *et al.* 2000; Schopf *et al.* 2008; McInerney *et al.* 2009; Morris *et al.* 2013). Current microbial population models fail to notice the empirical facts of the thermodynamically predicted ARP‐driven mutualism. Meanwhile, these models can successfully describe the population dynamics of phototrophic microorganisms and heterotrophic aerobic bacteria that generate energy from aerobic respiration (Monod 1950; Grovar 1997). In a previous study, we analysed a population growth model incorporating the ARP effect and confirmed that it exhibits qualitatively different behaviours from those observed in traditional microbial population growth models when the availability of catabolic energy is low, which is unusual for chemotrophs in aerobic conditions but typical for in anaerobic conditions. Introducing thermodynamics into population ecology would offer researchers a theoretical basis for understanding the ecology of chemotrophic microorganisms inhabiting anaerobic conditions, such as the deep subsurface, where chemotrophic microorganisms comprise the bulk of biomass and ~ 15% of the total biomass in the biosphere (Bar‐On *et al.* 2018).

In the present report, we develop a simple mathematical model for a mutualistic interaction in which the catabolic by‐product of the first species is utilised as a catabolic resource by the second species. Ecological models have developed largely in two ways: (1) to find out fundamental laws that help us to tease out complex biological aspects and interactions (simple mathematical models referred to by Scheffer & Carpenter (2003)) or (2) to build predictive and explanatory models for ecological consequences (large simulation models referred to by Scheffer & Carpenter (2003)). To highlight the importance and advantage of considering ARP that has been neglected in theoretical ecology, we focused on the problem in the simplest possible cases. Although a number of complications necessary for realistic geochemical applications of the model have been neglected at this stage of development, this simplification allows a transparent presentation of the essential ideas of the model, and a rigorous mathematical analysis. Here, we discuss how the consumption of the by‐product of species by others reduces the minimum energy demand for persistence, expands the realised niche and boosts material flow through ARP effect.

## The model for two chemotrophs catalysing the sequential catabolic reactions

In this analysis, we specifically consider two microbes where the catabolic by‐product of the first species is used as a catabolic resource by the second. For simplicity, they are referred to as Species 1 and 2 in this paper; in practice, functional groups could be used instead of species, with each functional group potentially including multiple species of microbes with a similar function in catalysing a catabolic reaction. Species 2 utilises as a resource the by‐product of a catabolic reaction produced by Species 1. Let *x*_1_ and *x*_2_ be the densities of Species 1 and 2, *y* be the concentration (in mol L^−1^) of the resource utilised by Species 1, and *z* be the concentration of the resource utilised by Species 2. As an example, consider the nitrifying bacteria in Fig. 1, which utilise the following energy‐harvesting reactions:

(3a) $${\text{NH}}_{4}^{+}+1.5O_{2}\to {\text{NO}}_{2}^{-}{\;+\;H}_{2}O\;+\;2H^{+}\;-\Delta G^{\circ }=195\;\text{kJ}\;{\text{mol}}^{-1}$$

(3b) $${\text{NO}}_{2}^{-}+0.5O_{2}\to {\text{NO}}_{3}^{-}\;-\Delta G^{\circ }=74.1\;{\text{kJ mol}}^{-1}$$

where −Δ*G*º denotes the negative change in the standard Gibbs energy of reaction (in kJ mol^−1^), indicating a reaction‐specific energy acquisition per reaction without the ARP effect, and all values of ∆*G*° reported in this paper are values at 273.15 K and 1 atm (Box 1 and Fig. 2a). In this case, *y* is the concentration of NH_4_^+^ (the resource used by Species 1) and *z* is the concentration of ${\text{NO}}_{2}^{-}$ (the resource used by Species 2). The abundance of all existing chemical substances and environmental factors especially temperature and pressure affect the activities of *y* and *z*. Because our primary objective is to build a simple mathematically tractable model, the activity coefficients are assumed to be unity, and the concentrations of other materials are constant, for example the concentrations of other materials are insensitive to the progress of reactions (3a) and (3b) because of the existence of some buffering capacity.

In a previous paper (Seto & Iwasa 2019), we studied the population dynamic outcomes of ARP effect in a single species of microbe with changes in the ratio of catabolic resource to by‐product of its environment as the energy‐harvesting reaction proceeds. Based on our previous arguments, the population growth rate of microbes is constrained by the energy they can obtain from catabolic reactions, referred to as oxidation–reduction (redox) reactions (Box 1). A set of differential equations can be derived for *x*_1_, *x*_2_, *y* and *z*, as follows:

(4a) $$\frac{dx_{1}}{dt}=q_{1}\left\{c_{1}r_{1}\frac{y}{K_{y}+y}\left(-\Delta G_{1}^{{}^{\circ }}+RT\ln\frac{y}{\alpha_{1}z^{n}}\right)-m_{1}\right\}x_{1}$$

(4b) $$\frac{dx_{2}}{dt}=q_{2}\left\{c_{2}r_{2}\frac{z}{K_{z}+z}\left(-\Delta G_{2}^{{}^{\circ }}+RT\ln\frac{z}{\alpha_{2}}\right)-m_{2}\right\}x_{2}$$

(4c) $$\frac{dy}{dt}=I_{y}-r_{1}\frac{y}{K_{y}+y}x_{1}-D_{y}y$$

(4d) $$\frac{dz}{dt}=I_{z}+nr_{1}\frac{y}{K_{y}+y}x_{1}-r_{2}\frac{z}{K_{z}+z}x_{2}-D_{z}z$$

where *q_i_* is the biomass yield of Species *i* for a given energy gain, *c_i_* is the fraction of useful energy (0 < *c* < 1) excluding energy expenditure such as loss by heat transfer, *r_i_* is the maximum catalytic rate per unit of biomass and *m_i_* is the maintenance energy. *K_y_* and *K_z_* are the Michaelis–Menten coefficients for *y* and *z*, respectively, *I_y_* and *I_z_* are the influxes and *D_y_* and *D_z_* are diffusion rates for *y* and *z*, respectively. The terms within braces in eqns (4a) and (4b) indicate the net energy acquisition per unit time, and the terms within brackets describe the energy acquisition per reaction given by eqns (2a) when *y* and *z* are used, respectively. −$\Delta G_{i}^{{}^{\circ }}$ is the negative change in the standard Gibbs energy when Species *i* utilises 1 mol of *y* (for *i* = 1) and *z* (for *i* = 2),* n* is the mole ratio between *y* and *z*, which corresponds to the mole number of produced *z* when *x*_1_ utilises 1 mol of *y*, and α_1_ and α_2_ are the ratios of by‐products to reactants aside from *y* and *z.* In eqns (3a) and (3b), α_1_ = ([H_2_O][H^+^]^2^)/[O_2_] and α_2_ = [NO_3_^‐^]/[O_2_]^0.5^. Definitions of the symbols, their units and default values are presented in Table S1.

Reaction of *y* and *z* can occur both biologically and non‐biologically. For example, the abiotic transformations of nitrogen from ammonia to nitrate via nitrite can proceed photochemically in the absence of nitrifiers (Doane 2017). Although the intensity of the competition between abiotic and microbial reactions depends on environmental factors (e.g. temperature, coexisting chemical substances, water availability and pH) (Melton *et al.* 2014; Liu *et al.* 2017), we have assumed that microbial reactions occur much faster than non‐biological reactions and so we have neglected the latter.

## Steady States of the Model

For a microbial population utilising a catabolic reaction that generates little energy, the time required to attain a steady state from a given population size would range from several days to years because of their slow growth (Hoehler & Jørgensen 2013). A steady‐state analysis serves as a guide to where the system is heading at any time. The steady states of the model can be obtained by setting eqns (4a–d) equal to zero. There are four possible steady‐state outcomes: neither species exists (*E*_0_), only Species 1 exists (*E*_1_), only Species 2 exists (*E*_2_) and both species coexist (*E*_3_). There exists at most one steady state in this system (Box 2).

Box 2 **Graphical analysis of the steady states**

**Figure 3:**
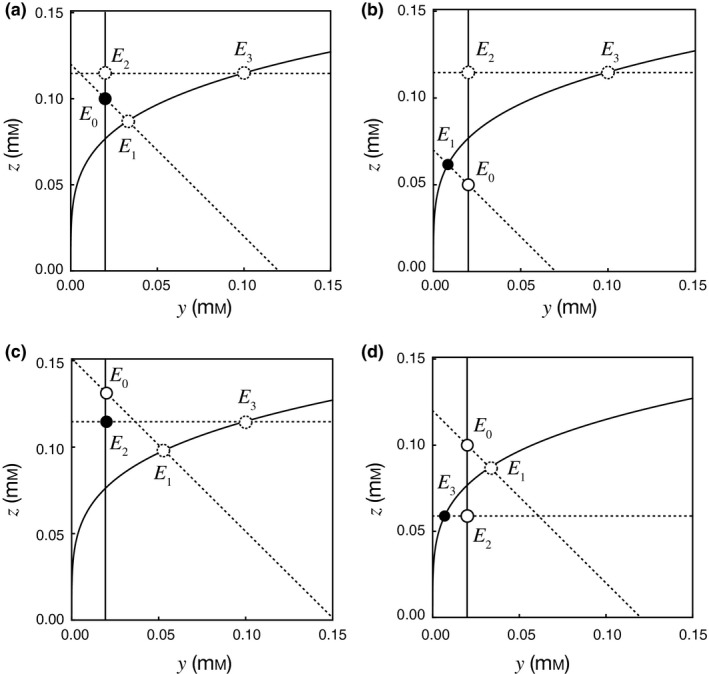
The existence and local stability of four possible steady states in the (*y*,*z*)‐plane. The curve given by eqn 5a, *I_y_*/*D_y_*, the curve given by eqn 5b and *nD_y_y* + *D_z_z* = *nI_y_* + *I_z_* are indicated by the solid curves, solid vertical lines, dotted horizontal lines and dotted lines with a negative slope, respectively. The labels for the circles indicate the four possible steady states: *E*_0_, without species; *E*_1_, with Species 1 only; *E*_2_, with Species 2 only and *E*_3_, with both species coexisting. The open and closed circles indicate unstable steady states and a single stable steady state, respectively. The dotted circles indicate that the condition for existence is not satisfied with these (*y*,*z*) values. Definitions of the symbols, their units and default values are presented in Table S1.

The rates of population growth of the two species, given by eqns (4a) and (4b), depend only on *y* and *z*. Plotting these functions on the (*y*, *z*)‐plane is useful for identifying all the steady states (Fig. 3).

In the steady state, d*x*_1_/d*t* = 0 and d*x*_2_/d*t* = 0. On the (*y*, *z*)‐plane, d*x*_1_/d*t* = 0 is represented by a curve corresponding to the net energy acquisition per unit time within braces to zero, which can be rewritten as:

(5a) $$z={\left[\frac{y}{\alpha_{1}}\exp\left\{\frac{1}{\mathrm{RT}}\left(-\Delta G_{1}^{{}^{\circ }}-\frac{m_{1}\left(K_{y}+y\right)}{c_{1}r_{1}y}\right)\right\}\right]}^{\frac{1}{n}},$$

which describes a curve with a positive slope. d*x*_1_/d*t* = 0 is also satisfied when *x*_1_ = 0; from eqn 4c, this results in a vertical line, *y* = *I_y_*/*D_y_*. These two curves are shown as solid lines in Fig. 3. The other condition, d*x*_2_/d*t* = 0, can be depicted as two straight lines on the (*y*, *z*)‐plane. The first is the horizontal line *z* = *constant*, which comes from setting the net energy acquisition per unit time within braces of *x*_2_ to zero and then rearranging eqn 4b to the following:

(5b) $$c_{2}r_{2}\frac{z}{K_{z}+z}\left(-\Delta G_{2}^{{}^{\circ }}+RT\ln\frac{z}{\alpha_{2}}\right)=m_{2}.$$

The second of the lines is *nD_y_y + D_z_z* = *nI_y_ + I_z_, *which is derived from eqns (4c) and (4d). This is a straight line with a negative slope. These two lines are shown as broken lines in Fig. 3. In the steady state, both d*x*_1_/d*t* = 0 and d*x*_2_/d*t* = 0, so *y* and *z* can be identified from the intersections between the solid and broken lines. When the values of *y* and *z* are known, *x*_1_ and *x*_2_ can be calculated by setting eqns 4c and 4d equal to zero. In this way, all the steady states related to eqn 4 can be identified.

Figure 3 illustrates four cases in which one of the four types of steady state (*E*_0_, *E*_1_, *E*_2_ and *E*_3_, shown on each diagram by the labelled circles) is stable. The population size of a species present in the ecosystem needs to be positive for conditions *E*_1_, *E*_2_ and *E*_3_. This can be established from the relative positions of the circles and the lines in Fig. 3, as explained in Appendix S2. For example, *x*_1_ for *E*_1_ is positive when the point labelled *E*_0_ is below the curve for eqn 5a and negative when *E*_0_ is above the curve. Similarly, *x*_2_ for *E*_2_ is positive when *E*_0_ is above the horizontal broken line for eqn 5b and negative when *E*_0_ is below this line. Both *x*_1_ and *x*_2_ are positive for *E*_3_ when the point *E*_3_ is to the left of the vertical line and below the negatively sloping broken straight line.

In Fig. 3, the stable steady state is indicated by a closed circle and the unstable steady states by open circles. The local stability of these steady states can be established from the Jacobian matrix for the corresponding steady state. According to the analysis in Appendix S2, the system always has a single stable steady which is globally stable, to which all trajectories that start with *x*_1_ > 0 and *x*_2_ > 0 converge.

As before, the steady states that are stable can be established from the relative positions of the circles and lines in Fig. 3 (as explained in Appendix S2). *E*_0_ is the stable steady state of the system when it is above the solid curve for eqn 5a and below the broken horizontal line. *E*_1_ is the stable steady state when *E*_0_ is below the curve and *E*_1_ is below the broken horizontal line. *E*_2_ is the stable steady state when *E*_0_ is above the broken horizontal line and *E*_2_ is above the curve for eqn 5a. The coexistence steady state *E*_3_ is stable when *E*_3_ is to the left of the vertical line and below the broken straight line with a negative slope.

According to numerical analyses of eqn 4, starting from different initial values of positive *x*_1_ and *x*_2_, the system always converges to the same steady state. We never observed a perpetual cycle, chaotic fluctuation or bistability. This suggested that there exists a single steady state that is globally stable (Appendix S1). The results of mathematical analyses are summarised in Box 2.

## Effect of the ARP

In this section, we examine the effect of the ARP on the population dynamics.

### *Reduced minimum* −∆*G*° *for successful invasion*

From eqns (4a) and (4b), we can calculate the minimum −∆*G*°, the energy production without the ARP effect, required for a species invasion. In general, a higher minimum −∆*G*° value requires a species to harness an energetically more favourable reaction (with higher −$\Delta G_{i}^{{}^{\circ }}$) to survive. There are the minimum −∆*G*° values for two cases: (1) when a single species attempts to invade an empty system and (2) when the species attempts to invade a system occupied by the other species. Let θ*_i,solo_* and θ*_i,acc_* be the minimum −∆*G*° values required for successful invasion in cases (1) and (2), respectively (with the subscripts *solo* and *acc* indicating that the invading species *i* is alone or accompanied by other species) (Appendix S3). The difference between θ*_i,solo_* and θ*_i,acc_* is an indication of the effect of the ARP arising from the presence of the other species. If the invasion condition for a species is satisfied more easily in the presence of the other species, this indicates that the presence of the other species benefits the focal species either by preventing its extinction or by helping to increase its population if rare. If both species benefit from the presence of each other with regard to the invasiveness when rare, this indicates there is mutualism between the two species (see Appendix S4 for the definition of mutualism in this study).

**Figure 4:**
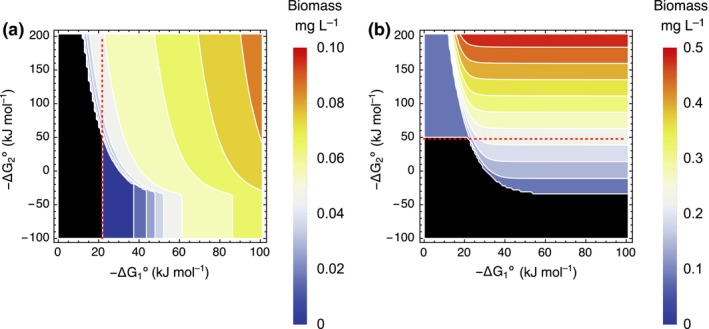
The steady‐state densities of (a) Species 1 and (b) Species 2. The horizontal and vertical axes represent −$\Delta G_{1}^{{}^{\circ }}$ and −$\Delta G_{2}^{{}^{\circ }}$. The red dashed line shows the minimum value of −$\Delta G_{i}^{{}^{\circ }}$ for the successful invasion for species *i* when the invading species i is alone. The black region indicates the vacant niche where the species becomes extinct. The steady‐state biomasses of Species 1 and 2 were calculated with the other parameters fixed. The values of those parameters are listed in Table S1.

In Appendix S3, we show that θ_1_ *_,acc_ < *θ_1_ *_,solo_*, implying that the presence of Species 2 helps the invasion of Species 1 or helps Species 1 avoid extinction. We also show that θ_2_ *_,acc_ < *θ_2_ *_,solo_*, which implies that the minimum −$\Delta G_{2}^{{}^{\circ }}$ required for the successful invasion of Species 2 is smaller in the presence of Species 1. Next, consider the contribution of the ARP. Even without the ARP, θ_2_ *_,acc_ < *θ_2_ *_,solo_*, but the ARP increases the magnitude of the difference between θ_2_ *_,acc_* and θ_2_ *_,solo_*. More importantly, θ_1_ *_,acc_* is smaller than θ_1_ *_,solo_* only when the ARP contributes to the energy acquisition per reaction of Species 1. Fig. 4 shows the steady‐state responses of the densities of Species 1 and 2 to the combination of −$\Delta G_{1}^{{}^{\circ }}$ and −$\Delta G_{2}^{{}^{\circ }}$. The ARP can provide an opportunity for Species 1 to utilise a low energy reaction (with small −$\Delta G_{i}^{{}^{\circ }}$) to invade a system with a second species that removes the by‐product of the energy‐harvesting reaction of Species 1. These results suggest a mutualistic interaction between the two species. The biogeochemical importance of this will be discussed later.

### Expanded niche

The environmental condition in which a species can survive is called an ecological niche. Typically, this can be represented as a region of a plane where the axes represent physical (non‐biological) conditions. Fundamental niche is the entire set of conditions under which an organism can live in the absence of others. In reality, species interact with competitors, parasites, pathogens, etc.; thus, the niche is affected by the presence of these species. The latter is called realised niches (Hutchinson 1957).

Because Species 1 supplies the resources for Species 2, its presence expands the conditions for Species 2’s survival, and hence the realised niche of Species 2. This is a standard effect, known as ‘niche construction’ or ‘niche changing’ (Erwin 2008; Laland *et al.* 2016). In our model, the ARP term increases the fitness of Species 1 because the presence of Species 2 reduces the abundance of the by‐product of Species 1, resulting in the expansion of the realised niches of Species 1. Hence, both species expand their realised niches in the presence of the other species and are able to live in conditions in which they would not otherwise survive.

In the case illustrated in Fig. 4b, Species 2 can survive alone only when it utilises a reaction with −$\Delta G_{2}^{{}^{\circ }}$ > 47.6 kJ mol^−1^. It must use the material available for metabolism under this constraint. When Species 2 is present, the condition for the survival of Species 1 expands because the value of −∆*G*° required for it to be able to invade is reduced. Species 1 may then be able to adopt a different material as its resource that is energetically less favourable than the original resource (e.g. iron instead of manganese in Fig. 2a). This would result in different by‐products that can be used by other species.

**Figure 5:**
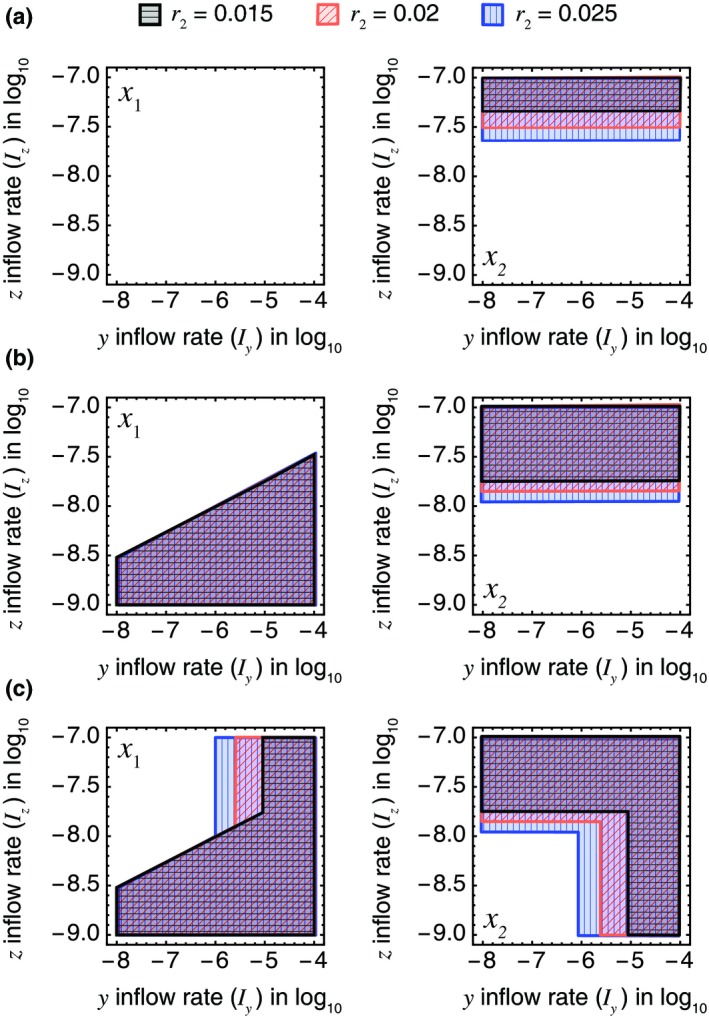
The fundamental niches (a and b) and realised niches (c) of Species 1 (left panels) and Species 2 (right panels). The horizontal and vertical axes represent the inflows *I_y_* and *I_z_* in units of mmol L^−1^ h^−1^. The black, red and blue curves indicate the boundaries for three values of the maximum catalytic rate of Species 2, *r*_2_ (0.015, 0.02 and 0.025 mmol h^−1^ g^−1^, respectively). The abundant resource premium has been ignored in (a) (i.e. −∆*G_i_* = −$\Delta G_{i}^{{}^{\circ }}$) but taken into consideration in (b) and (c). The steady‐state biomasses of the two species were calculated with the other parameters fixed. The values of those parameters are listed in Table S1.

Figures 5a and b show the fundamental niches of Species 1 and 2 relative to the inflows *I_y_* and *I_z_* (mmol L^−1^ h^−1^). The curves indicate the boundaries for different values of *r*_2_ the maximum catalytic rate of Species 2. Figure 5a shows the case when the ARP was ignored (i.e. −∆*G* = −∆*G*°), whereas the ARP was taken into consideration in Fig. 5b. To highlight the effect of the ARP on the niche of Species 1, the parameters were chosen to ensure that Species 1 could not grow with any combination of *I_y_* and *I_z_* in the absence of the ARP. Irrespective of whether the ARP is considered, the increase in *r*_2_ increases the ability of Species 2 to invade, allowing it to invade the system with smaller *I_z_.* Although the ARP results in the expansion of the fundamental niches of Species 1 and 2 for this particular parameter set, with different parameter values the ARP may lead to a reduction in the species’ fundamental niches. However, because the ARP brings a benefit to each species in the presence of the other species, the increase in the ability of Species 2 to invade the system always expands the realised niches of both species (Fig. 5b and c). Appendix S5 examines how the invasiveness of the species depends on the parameter values.

### Increased biomass

**Figure 6:**
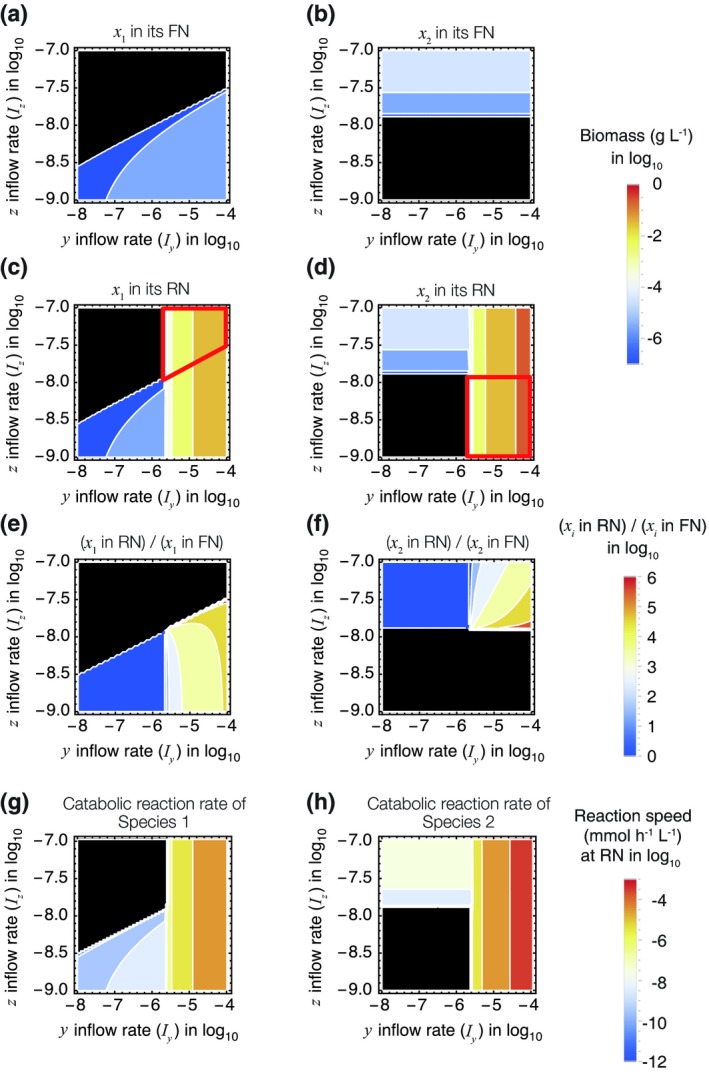
The steady‐state biomass (a–d) and the increased biomass and speed of catabolic reactions in the presence of the mutualistic partner species (e–h). The horizontal and vertical axes represent the inflows *I_y_* and *I_z_* in units of mmol L^−1^ h^−1^ (a, b) The biomass of Species 1 or 2, respectively, invades an empty system, which correspond to the biomass of Species 1 or 2 in its fundamental niche (FN). (c, d) The biomass of Species 1 or 2, respectively, invades a system in the presence of the other species, which correspond to the biomass of Species 1 or 2 in its realised niche (RN). The black region indicates the vacant niche in which a species becomes extinct. (e, f) The increased steady‐state biomasses of Species 1 and 2, respectively, resulting from the presence of the mutualistic partner (The ratio of the biomass at its RN to FN). (g, h) The increased catabolic reaction rates of Species 1 and 2, respectively, resulting from the presence of the mutualistic partner. The steady‐state values were calculated with the other parameters fixed at the standard values shown in Table S1.

Figures 6a–d illustrate the steady‐state biomasses of Species 1 and 2 in response to the combination of *I_y_* and *I_z_* (mmol L^−1^ h^−1^) when *r_2_* = 0.02 mmol h^−1^ g^−1^ (shown as the red shaded region in Fig. 5b and c). In Fig. 6a and b, the coloured region indicates the combinations of parameters for which a species can invade an empty system where no organisms exist (i.e. for condition *E*_0_) while maintaining a positive biomass. They indicate the fundamental niches (FN) of the two species shown in Fig. 5b. However, these species can invade a system occupied by the other species when the parameters are within the region surrounded by the red lines in Fig. 6c and d; these show the realised niches (RN), which are larger than the fundamental niche of the focal species.

In Appendix S2, we demonstrate that when a species is able to successfully invade a system occupied by the other species, the steady‐state biomass of that species is always higher in the presence of the other species than in its absence. This difference can be considerable, potentially several orders of magnitude higher, as illustrated in Fig. 6e and f.

### Increased material flow

An argument similar to the one developed for biomass applies to the fluxes of *y* and *z* in the steady state (Appendix S6). The reaction speeds of catabolising *y* to *z* and *z* to its by‐products by the two species are always faster in the presence of the mutualistic partner than in its absence (Fig. 6g and h). Thus, ARP‐driven mutualism expands the possible material flow in a system. An ecosystem can be regarded as a combination of a biogeochemical network of materials and a network of interactions within communities (Naeem *et al.* 2012). Material–energy flow between different pools of chemical elements created by the organisms provides a framework for the network complex (Olff *et al.* 2009; Loreau 2010). The results of our study, however, imply that when organisms act as catalysts but do not pool the chemical elements of their energy‐harnessing reactions, this might alter the material flows of the ecosystem, with ARP‐driven mutualism further enhancing those flows. This could provide an important insight into coupling biogeochemical pathways with catabolic interactions among species to help understand the role of biodiversity in the functioning of ecosystems.

## Discussion

To survive, grow and reproduce, chemotrophic microbes need to synthesise biomass. They do this using energy obtained from a set of chemical reactions that convert a resource to a by‐product. The amount of energy obtained per reaction increases with the abundance of the resource and decreases with the abundance of the by‐product, the ARP effect (Seto & Iwasa 2019). The present study examined the interspecific mutualistic interaction resulting from this effect, its implications for population dynamics and its importance for expanding realised niches, boosting material flow through the ecosystem, and generating mutualistic interactions among the species in the ecosystem.

### The ARP results in increased non‐feeding interactions in microbial communities

Our analysis demonstrated that when a catabolic by‐product of the energy‐harvesting reaction of a species is utilised by a second species, this can improve the energy‐extraction efficiency of the first species because of the ARP effect, resulting in a mutualistic relationship between the two species. This report presents the first theoretical demonstration of this ARP‐driven mutualism.

The mutualism that occurs during syntrophy, a facet of symbiosis, can traditionally be defined as the cooperative cross‐feeding of multiple microbial species in the step‐by‐step degradation of a complex carbon substrate (Morris *et al.* 2013): a species degrades a carbon substrate as its carbon source and releases a by‐product, which is used by a second species as its carbon source, with the second species commensal on the first species. Non‐classical syntrophy refers to ARP‐driven mutualism rather than commensalism. The first confirmation of ARP‐driven mutualism in a mixed microbial culture was for a culture of *Methanobacillus omelianskii* (Baker 1939), in which strain S converted ethanol into acetate and hydrogen gas, and *Methanobacterium* strain M.o.H. then converted the hydrogen gas into methane. Since the value of −∆*G*° for the energy‐harvesting reaction of strain S is −19 kJ mol^−1^, this reaction could not feed strain S without the ARP effect (Bryant *et al.* 1967). Strain S was able to grow in the presence of its hydrogen‐removing partner when the hydrogen partial pressure was maintained at a sufficiently low level (< 100 Pa) (Schink 1997). Another example involves marine archaeal–bacterial consortia comprising anaerobic methane‐oxidising archaea and sulphate‐reducing bacteria (Hoehler *et al.* 1994; Boetius *et al.* 2000). The anaerobic methane‐oxidising archaea grow in a dense aggregate surrounded by the sulphate‐reducing bacteria (Boetius *et al.* 2000), utilising the following overall reaction:

(6a) $${\text{CH}}_{4}+{\text{SO}}_{4}^{2-}\to {\text{HCO}}_{3}^{-}+{\text{HS}}^{-}+H_{2}O\;-\Delta G^{\circ }=16{\;\text{kJ mol}}^{-1}.$$

How the energy‐harvesting reactions of these archaea and bacteria combine remains under debate, but one possible example is as follows (Konhauser 2007):

(6b) $${\text{CH}}_{4}+3H_{2}O\to 4H_{2}+{\text{HCO}}_{3}^{-}+H^{+}\;-\Delta G^{\circ }=-136{\;\text{kJ mol}}^{-1},$$

(6c) $$4H_{2}+{\text{SO}}_{4}^{2-}\to {\text{HS}}^{-}+{\text{OH}}^{-}+3H_{2}O-\Delta G^{\circ }=152{\;\text{kJ mol}}^{-1}.$$

If this combination is correct, the methane‐oxidising archaea would not be able to grow in the absence of the positive effect from ARP because of the negative value of −∆*G*° of eqn 6b. The rapid consumption of hydrogen gas by the sulphate‐reducing bacteria may allow anaerobic methane‐oxidising archaea to grow in the region neighbouring the sulphate‐reducing bacteria.

ARP‐driven mutualism is an example of a non‐trophic interaction that has been ignored in traditional ecological models. Early ecological network models were developed based on trophic interactions (May 1973; McCann *et al.* 1998; Milo 2002), but models over the last two decades have gradually included non‐trophic interactions, such as interference competition, mutualism, exploitation, commensalism and amensalism (Arditi *et al.* 2005; Goudard & Loreau 2007; Kéfi *et al.* 2012). The consequences of the ARP effect can be ignored in traditional ecological network models, which mainly comprised plants that harnessed light or animals that harnessed aerobic reactions with large −∆*G*°. However, the ARP effect may significantly alter microbial communities, especially those in the subsurface realm, which utilise reactions with small −∆*G*°. Because the ARP effect is generated by the bias of materials, its magnitude can be affected not only by microbial activity but also by physicochemical activity, such as the adsorption, diffusion and sedimentation of materials.

Although we focused on the ARP effect on the interaction type (from (0, +) to (+, +)), which arises based on material flows among catabolic processes (red solid arrows in Fig. 1), the material flows among anabolic processes (blue dotted arrows in Fig. 1) also alter the interaction type. For example, differences in the nutritional demands of C, N and phosphorus (P) to generate biomass (ecological stoichiometry) or for nutrient recycling influence such flows and, consequently, affect interaction types and community diversity (Loreau 1998, 2001; Loladze *et al.* 2004; Elser *et al.* 2012). Thus, a combination of geochemistry, thermodynamics and ecology is key to understanding the microbial biodiversity and ecosystem functioning.

### *Interspecific interactions among microbes affect the minimum* −∆*G*° *for invasion*

We demonstrated that ARP‐driven mutualism can reduce the minimum value of −∆*G*° needed for the successful invasion of both species. Estimates of −∆*G* values for various possible energy‐harvesting reactions that can take place in an environment have been used to predict the potential existence in those environments of microbes that utilise those reactions. For example, it was predicted in 1977 that there would be species that can harness a reaction that utilises nitrate and ammonia, the so‐called anammox reaction because the value of −∆*G* for the reaction is positive (Broda 1977). The existence of bacteria that harness the annamox reaction was confirmed almost 20 years later (Strous *et al.* 1999). Conversely, when a reaction has −∆*G*° < 0, it is generally considered that it would be unlikely to be utilised for microbial catabolic reactions because such reactions cannot produce energy when −∆*G* < 0.

In the fields of geochemistry and microbiology, experimental and theoretical approaches have been applied to establish the minimum energy requirement of cells based on physicochemical and biochemical factors (Hoehler 2004; Price & Sowers 2004; Heijnen 2010). Our analysis, however, demonstrated that interspecific microbial relationships can reduce the minimum −∆*G*° value needed for successful invasion, possibly resulting in mutualistic interactions of microbial species linked by material flows in the ecosystem. Thus, ecological factors should be taken into account when considering the minimum energy requirement of a cell for growth.

### ARP affects microbial evolution and ecosystem development

There are several mechanisms that can allow the ARP‐driven mutualism to evolve in a way that avoids the risk of one species being exploited by the other species and the subsequent collapse of the mutualism.

First, the spatial aggregation of cells can robustly maintain the ARP‐driven mutualism. Because the diffusion rate of a catabolic by‐product becomes lower as microbial aggregates are condensed, aggregate formation tends to negatively influence the growth of the microbe producing the by‐product but provides a great opportunity for a mutualistic species to exploit the by‐product while increasing the fitness of the partner species. Suppose that a mutant of the first microbial species produces the catabolic by‐product in a form that is easier for the second species to use than the wild‐type microbe’s by‐product. As a result, the mutant may enjoy higher fitness than the wild type and replace it. This requires a spatial structure in which the mutant microbes are collocated together with other mutant microbes, as commonly occurs in microbial mats or the marine archaeal–bacterial consortia discussed in section 5.1. In addition, ammonia‐ and nitrite‐oxidising bacteria that catalyse eqn 3a and b were confirmed to occur in clusters and frequently be in contact with each other within sludge flocs (Mobarry *et al.* 1996).

Second, increasing the mole number, *n*, of the by‐product of a species (*z* in our model) might be beneficial not only to other species that utilise this by‐product but also to the species itself (Appendix S5). When a species rapidly produces the by‐product of its energy‐harvesting reaction (high *n*), this can be detrimental to that species because it increases the concentration of the by‐product in the ecosystem. However, as shown in eqn 4a, the energy acquisition per reaction increases with increasing *n* (note that the molar concentration should be < 1) when the activities of the reactants and products are unchanged; this is not simply possible because the change in stoichiometric coefficient should be associated with the alteration of the form of the by‐product and the reaction speed, but may potentially favour a mutant species that can produce a greater amount of the by‐product relative to the reactant.

Third, the enlarged biomass may allow a mutant species with a high resource‐utilising ability to avoid local extinction. In traditional microbial population models, the steady‐state population density usually increases and converges to a particular level as the resource‐utilising ability increases (i.e. either the maximum catalytic rate per unit of biomass of species *i*, *r_i_*, increases, or *K_y_* and *K_y_*, the Michaelis–Menten constants for *y* and *z*, decrease). In our previous paper (Seto & Iwasa 2019), we demonstrated that the steady‐state microbial population density when the ARP effect has a significant influence decreases as the microbe’s resource‐utilising ability increases. This is because a microbial population with a very high resource‐utilising ability depletes resource*s*, reducing the energy available from the reaction and therefore its own growth rate, which eventually results in a decrease in the population size. A similar tendency was observed in the current model (Fig. S2). If several species that harness the same reaction coexist within a system, the species with the highest resource‐utilising ability will tend to exhibit the fastest growth rate and so will become dominant in the system. A mutant species with a higher resource‐utilising ability than the other species in the system would be favoured and would finally exist at low density, prone to local extinction (Matthies *et al.* 2004). However, if the microbial species has a mutualistic partner in the ecosystem, its steady‐state population will still decrease with increasing resource‐utilising ability, but it will be maintained at a higher level than when there is no mutualistic partner. Thus, ARP‐driven mutualism may act to reduce the risk of local extinction.

This process could result in the evolution of microbes that increase the speed of material flow in the ecosystem, and thus enhance ecosystem development. In the presence of the ARP effect, microbial species will tend to evolve in ways that increase the speed of material flows, resulting in an ecosystem composed of species tightly linked with each other by these material flows. In addition, the ARP effect could increase the mobility of materials that are energetically less favourable, which are less likely to be utilised by microbes than those that are energetically more favourable. ARP‐driven mutualism adds energetic value to these materials and may enhance the material cycle, which could easily cease without the ARP‐driven mutualism. This mechanism may have been of especial importance in the era before the so‐called Great Oxidation Event because, under anaerobic condition, the ARP can often have a significant influence on the energy acquisition per reaction. This possibility warrants detailed theoretical study from the viewpoint of the early evolution of life on Earth.

## Authorship

MS and YI designed research, performed research and wrote the paper.

## Data Availability

No new data was used.
