# Supplementary material for: The fitness of chemotrophs increases when their catabolic by‐products are consumed by other species
Source: Ecol Lett. 2019 Oct 14;22(12):1994–2005. doi: 10.1111/ele.13397 (PMC6899997; doi:10.1111/ele.13397)

Figure S1 Seto and Iwasa

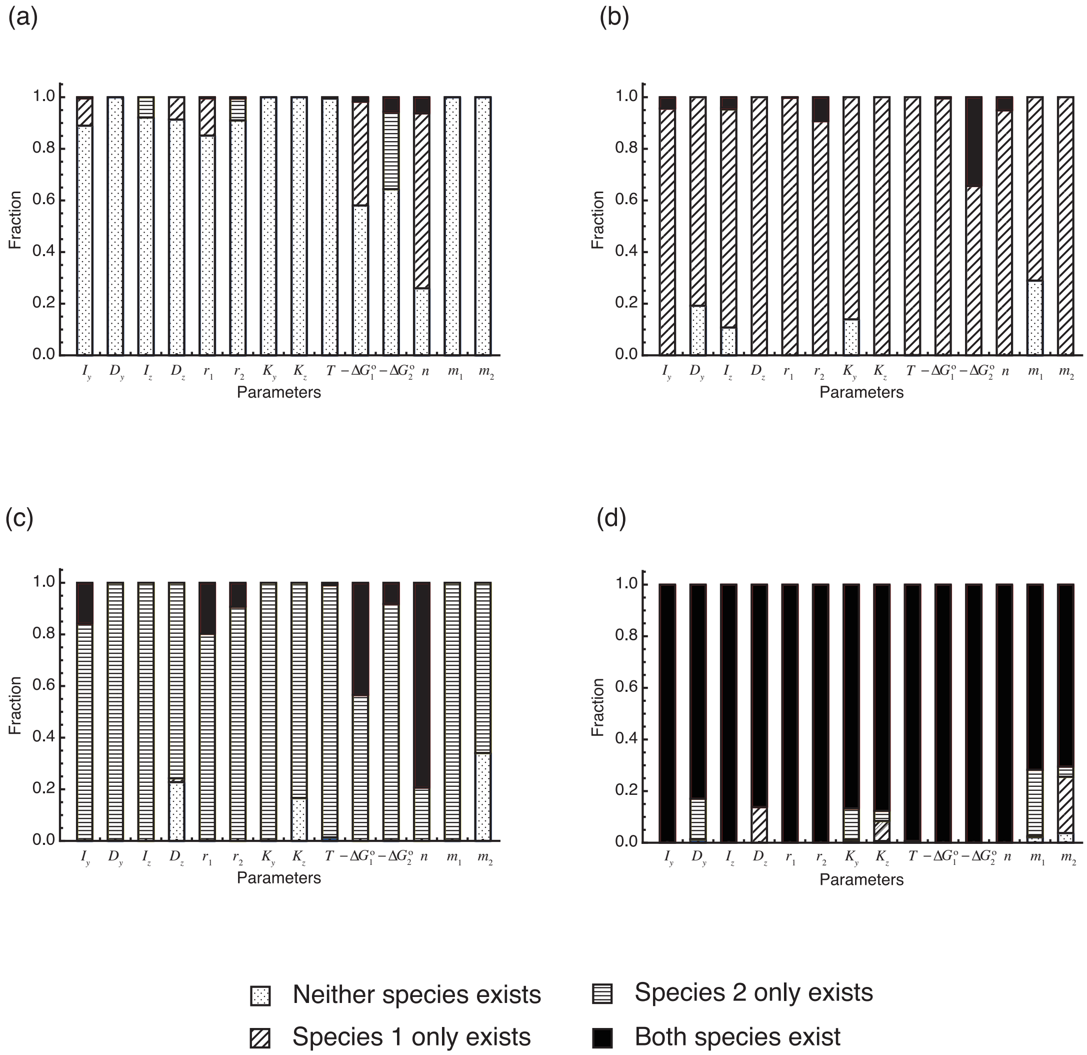

Figure S2 Seto and Iwasa

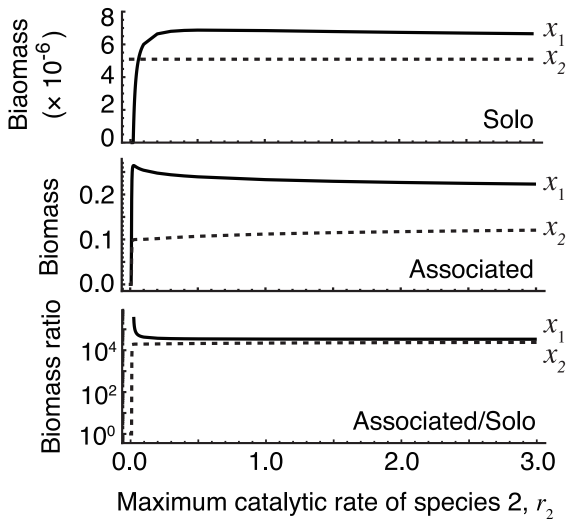

Supplement: Supplementary file 1 [file ELE-22-1994-s001.pdf]
