# Supplementary material for: The fitness of chemotrophs increases when their catabolic by‐products are consumed by other species
Source: Ecol Lett. 2019 Oct 14;22(12):1994–2005. doi: 10.1111/ele.13397 (PMC6899997; doi:10.1111/ele.13397)
Supplement: Supplementary file 2 [file ELE-22-1994-s002.docx]

| Symbols | Definition | Unit | Default value | Range |
| --- | --- | --- | --- | --- |
| $R$  $q_{i}$  $c_{i}$  $r_{1}$  $r_{2}$  $K_{y}$  $K_{z}$  $-\Delta G_{1}^{^{\circ}}$  $-\Delta G_{2}^{^{\circ}}$  $\alpha_{1}$  $\alpha_{2}$  $T$  $n$  $m_{1}$  $m_{2}$  $I_{y}$  $I_{z}$  $D_{y}$  $D_{z}$ | Gas constant  Amount of biomass that can be reproduced for a given energy gain of species *i*  Fraction of useful energy of species *i*  Maximum catalytic rate per unit of biomass of species 1  Maximum catalytic rate per unit of biomass of species 2  Michaelis-Menten constant for *y*  Michaelis-Menten constant for *z*  Standard Gibbs energy of the energy-harnessing reaction of species 1  Standard Gibbs energy of the energy-harnessing reaction of species 2  Ratios of by-products/ reactants aside from *y* and *z* of the energy-harvesting reaction of species 1  Ratios of by-products/ reactants aside from *y* and *z* of the energy-harvesting reaction of species 2  Absolute temperature  The mole ratio between $y$ and $z$  Maintenance energy per unit of biomass of species 1  Maintenance energy per unit of biomass of species 2  Inflow rate of $y$  Inflow rate of $z$  Outflow rate of $y$  Outflow rate of $z$ | kJ mol^-1^ K^-1^  g kJ^-1^  no dimension  mmol h^-1^ g^-1^  mmol h^-1^ g^-1^  mmol L^-1^  mmol L^-1^  kJ mol^-1^  kJ mol^-1^  *  *  K  No dimension  kJ h^-1^ g^-1^  kJ h^-1^ g^-1^  mmol L^-1^ h^-1^  mmol L^-1^ h^-1^  h^-1^  h^-1^ | 8.314 × 10^-3^  0.01  1  0.001  0.02  10^-6^  0.1  15  30  10^-8^  10^-9^  288.15  4  10^-4^  10^-4^  10^-7.3^  10^-8^  10^-5^  10^-5.8^ | 10^-4^ – 10^-1^  10^-4^ – 10^-1^  10^-6^ – 10^-1^  10^-6^ – 10^-1^  -100 – 100  -100 – 100  -20 – 80  10^-6^ – 4  10^-7^ – 10^-4^  10^-7^ – 10^-4^  10^-8^ – 10^-4^  10^-8^ – 10^-4^  10^-8^ – 10^-4^  10^-8^ – 10^-4^ |

Table S1. Symbols, definition, units, default values, and ranges of parameters

* Units depend on the by-products and reactants involved in the energy-harvesting reaction
