# Supplementary material for: The fitness of chemotrophs increases when their catabolic by‐products are consumed by other species
Source: Ecol Lett. 2019 Oct 14;22(12):1994–2005. doi: 10.1111/ele.13397 (PMC6899997; doi:10.1111/ele.13397)
Supplement: Supplementary file 3 [file ELE-22-1994-s003.docx]

**Supporting Information**

**S1. Dynamic behavior of Eq. (4)**

A numerical analysis of the dynamics of Eq. (4) showed the following behavior:

[1] Steady state can occur with four outcomes: where neither species exists ($x_{1}=x_{2}=0$) ($E_{0}$); where only Species 1 exists ($x_{1}=\hat{x}_{1}>0, x_{2}=0$) ($E_{1}$); where only Species 2 exists ($x_{1}=0, x_{2}=\hat{x}_{2}>0$) ($E_{2}$); and where both species coexist ($x_{1}=x_{1}^{*}>0, x_{2}=x_{2}^{*}>0$) ($E_{3}$).

[2] Steady states may or may not exist, depending on the values of the parameters. However, if a steady state exists, it will be unique, that is, $\hat{x}_{1}$, $\hat{x}_{2}$, $x_{1}^{*}$, and $x_{2}^{*}$ are unique for a given set of parameter values.

[3] We did not observe any perpetual oscillation, chaotic fluctuation, or bistability. Supporting Information 2 examines Eqs. (4a–d) at steady state and the local stability conditions. This confirmed that, for a given set of parameters, only a single steady state satisfies the conditions for existence and local stability. A numerical analysis of the equations in Eqs. (4a–d) confirmed that it is globally stable.

**S2. Steady states and their local stability**

The stability of a steady state can be established from the eigenvalues of the Jacobian for the dynamics given by Eqs. (4a–d) around the focal steady state, which is as follows:

$J=\left( \begin{matrix} q_{1}F & 0 & F_{y}x_{1} & F_{z}x_{1} \\ 0 & q_{2}G & 0 & G_{z}x_{2} \\ -a & 0 & -b-D_{y} & 0 \\ na & -c & nb & -d-D_{z} \end{matrix} \right)$, (S.1)

where

$F=F\left( y,z \right)=c_{1}r_{1}\frac{y}{K_{y}+y}\left( g_{1}+RT\ln\frac{y}{\alpha_{1}z^{n}} \right)-m_{1}$,

$G=G(z^{*})=c_{2}r_{2}\frac{z}{K_{z}+z}\left( g_{2}+RT\ln\frac{z^{n}}{\alpha_{2}} \right)-m_{2}$,

$F_{y}=\frac{q_{1}c_{1}r_{1}}{\left( K_{y}+y^{*} \right)^{2}}\left\{ RT\left( K_{y}+y \right)+K_{y}\left( g_{1}+RT\ln\frac{y}{\alpha_{1}z^{n}} \right) \right\}$ ,

$F_{z}=-\frac{nq_{1}c_{1}r_{1RTy}}{\left( K_{y}+y \right)z}$ ,

$G_{z}=\frac{q_{2}c_{2}r_{2}}{\left( K_{z}+z \right)^{2}}\left\{ nRT\left( K_{z}+z \right)+K_{z}\left( g_{2}+RT\ln\frac{z^{n}}{\alpha_{2}} \right) \right\}$,

$a=r_{1}\frac{y}{K_{y}+y}$, $b=\frac{{r_{1}K}_{y}}{\left( K_{y}+y \right)^{2}}x_{1}$, $c=r_{2}\frac{z}{K_{z}+z}$, and $d=\frac{{r_{2}K}_{z}}{\left( K_{z}+z \right)^{2}}x_{2}$,

and $x_{1}$, $x_{2}$, $y$, and $z$ are the values in the steady state. $F\left( y,z \right)=0$ and $G\left( z \right)=0$ are identical to Eq. (5a) and Eq. (5b), respectively. $F\left( y,z \right)<0$ holds when the point $\left( y,z \right)$ is above the curve given by Eq. (5a) in Fig. 3. Conversely, $F\left( y,z \right)>0$ holds when the point $\left( y,z \right)$ is below the curve. Similarly, $G(z) > 0$ when the point $\left( y,z \right)$ is above the curve described by Eq. (5b), shown as the broken horizontal line in Fig. 3; and $G(z) < 0$ when the point $\left( y,z \right)$ is located below the broken horizontal line.

*S2.1. Existence and local stability of* $E_{0}=\left( 0, 0, y_{0}, z_{0} \right)$

From Eq. (4c) and (4d), $y_{0}={I_{y}}/{D_{y}}$ and $z_{0}={I_{z}}/{D_{z}}$. This steady state always exists. The characteristic polynomial for Eq. (A.1) at $E_{0}$ is

$\left( q_{1}F-\lambda\right)\left( q_{2}G-\lambda\right)\left( -D_{y}-\lambda\right)\left( -D_{z}-\lambda\right)=0$ . (S.2)

All the eigenvalues are negative if ${F(y}_{0},z_{0})<0$and $G(z_{0})<0$. in Fig. 3, $E_{0}$ is locally stable if $E_{0}$ is located above the curve and below the broken horizontal line (Fig. 3(a)).

*S2.2. Existence and local stability of* $E_{1}=\left( \hat{x}_{1}, 0,\hat{y}_{1}, \hat{z}_{1} \right)$

In Fig. 3, the point $\left( \hat{y}_{1}, \hat{z}_{1} \right)$ is given by the intersection of the curve and the broken straight line with a negative slope. Solving Eq. (4c) = 0 for $\hat{x}_{1}$ gives

$\hat{x}_{1}=\frac{\left( K_{y}+\hat{y}_{1} \right)}{r_{1}\hat{y}_{1}}(D_{y}\hat{y}_{1}-I_{y})$. (S.3)

$E_{1}$ exists if $\hat{y}_{1}<y_{0}$. Because ${nD}_{y}\hat{y}_{1}+D_{z}\hat{z}_{1}=nI_{y}+I_{z}$, $\hat{z}_{1}>z_{0}$ is satisfied when $\hat{y}_{1}<y_{0}$. When $\hat{y}_{1}<y_{0}$ and $\hat{z}_{1}>z_{0}$, $E_{1}$ is below the curve (Fig. 3(b)). Thus, in Fig. 3, a steady state for $E_{1}$ exists if the point $E_{0}$ is below the curve. When $E_{1}$ exists, $E_{0}$ is unstable because $F\left( y_{0},z_{0} \right)>0$, and $E_{2}$ is also unstable (as shown in section S1.4, below).

The characteristic polynomial for Eq. (B.1) at $E_{1}$ is

$\left( q_{2}G-\lambda\right)\left( \lambda^{3}+a_{1}\lambda^{2}+a_{2}\lambda+a_{3} \right)=0$ , (S.4)

where $a_{1}=b+D_{y}+D_{z}$, $a_{2}=bD_{z}+D_{y}D_{z}+{aF}_{y}\hat{x}_{1}-anF_{z}\hat{x}_{1}$, and

$a_{3}=aD_{z}F_{y}\hat{x}_{1}-a{nD}_{y}F_{z}\hat{x}_{1}$. When the Routh–Hurwitz criteria are applied, all the eigenvalues of $\lambda^{3}+a_{1}\lambda^{2}+a_{2}\lambda+a_{3}=0$ are positive because $a_{1}>0$, $a_{2}>0$, $a_{3}>0$, $a_{1}a_{2}-a_{3}>0$, and $a_{3}(a_{1}a_{2}-a_{3})>0$. Consequently, $E_{1}$ is locally stable if $G\left( \hat{z_{1}} \right)<0$, or if the point $E_{1}$ is located below the broken horizontal line in Fig. 3.

*S2.3. Existence and local stability of E_2_ =* $\left( 0, \hat{x}_{2}, \hat{y}_{2},\hat{z}_{2} \right)$

Because Species 1 is absent in condition *E*_2_, $\hat{y}_{2}=y_{0}=I_{y}/D_{y}$. In addition, $\hat{z}_{2}$ is identical to the broken horizontal line in Fig. 3. By solving Eq. (4d) for *x*_2_, substituting *x*_1_ = 0,

$\hat{x}_{2}=\frac{\left( K_{z}+\hat{z_{2}} \right)}{r_{2}\hat{z_{2}}}(D_{z}\hat{z}_{2}-I_{z})$ (S.5)

*E*_2_ exists if $\hat{z}_{2}< z_{0}$. When $\hat{z}_{2}<z_{0}$, *E*_0_ is located above the broken horizontal line in Fig. 3 (Fig. 3(c)). Thus, *E*_2_ exists if *E*_0_ is located above the broken horizontal line in Fig. 3. When *E*_2_ exists, *E*_0_ is unstable because $G\left( z_{0} \right)>0$.

The characteristic polynomial of Eq. (B.1) at *E*_2_ is

$\left( q_{1}F-\lambda\right)\left( -D_{y}-\lambda\right)\left( \lambda^{2}+\left( d+D_{z} \right)\lambda+cG_{z}\hat{x}_{2} \right)=0$ . (S.6)

All the eigenvalues are negative if $F(\hat{y}_{2},\hat{z}_{2})<0$. $E_{2}$ is locally stable if $F(\hat{y}_{2},\hat{z}_{2})<0$, or if the point $E_{2}$ is above the curve in Fig. 3(c). When $E_{2}$ is above the curve, $E_{0}$ on the broken straight line with a negative slope should be located left to *E*_1_; thus, $y_{0}< \hat{y}_{1}$ when the condition for existence of $E_{1}$ is not satisfied.

*S2.4. Existence and local stability of E_3_ =* $\left( x_{1}^{*}, x_{2}^{*}, y^{*}, z^{*} \right)$

For *E*_3_, $z^{*}=\hat{z}_{2}$ as $\left( y^{*}, z^{*} \right)$ is the intersection of the curve and the broken horizontal line in Fig. 3. Solving Eq. (4c) = 0 for $x_{1}^{*}$ gives

$x_{1}^{*}=\frac{\left( K_{y}+y^{*} \right)}{r_{1}y^{*}}(D_{y}y^{*}-I_{y})$ , (S.7)

and solving Eq. (4d) = 0 and substituting in Eq. (S.7) gives

$x_{2}^{*}=\frac{1}{r_{2}}\left( I_{z}+I_{y}n-{nD}_{y}y^{*}-D_{z}z^{*} \right)$.

$E_{3}$ exists if

$y^{*}<y_{0}$ and $z^{*}<n\left( \frac{I_{y-}D_{y}y^{*}}{D_{z}}-\frac{D_{y}}{D_{z}} \right)+\frac{I_{z}}{D_{z}}$ (S.8)

The point $E_{3}$ should be located to the left of the vertical line in Fig. 3 to satisfy the first condition, and below the broken straight line with a negative slope to satisfy the second condition (Fig. 3(d)). When $E_{3}$ exists, the condition for existence of $E_{0}$ is not satisfied, and the local stability conditions of $E_{1}$ and $E_{2}$ are not satisfied because the point $E_{1}$ is located above the broken horizontal line and $E_{2}$ is located below the curve in Fig. 3.

The characteristic polynomial for Eq. (S.1) at $E_{3}$ is

${\lambda^{4}+a_{1}\lambda}^{3}+a_{2}\lambda^{2}+a_{3}\lambda+a_{4}=0$ (S.9)

where

$a_{1}=b+d+D_{y}+D_{z}$,

$a_{2}=bd+dD_{y}+{bD}_{z}+{D_{y}D}_{z}+aF_{y}x_{1}^{*}-anF_{z}x_{1}^{*}+cG_{z}F_{y}x_{2}^{*}$,

$a_{3}=adF_{y}x_{1}^{*}+dD_{z}F_{y}x_{1}^{*}-an{D_{y}F}_{z}x_{1}^{*}+bcG_{z}x_{2}^{*}+{{cD}_{y}G}_{z}x_{2}^{*}$ ,

$a_{4}=acF_{y}G_{z}x_{1}^{*}x_{2}^{*}$ .

By applying the Routh–Hurwitz criteria, it can be shown that all the eigenvalues of Eq. (S.9) are positive because $a_{1}>0$, $a_{2}>0$, $a_{3}>0$, $a_{4}>0$, $a_{1}a_{2}-a_{3}>0$, $a_{3}(a_{1}a_{2}-a_{3})>0$, and $a_{3}\left( a_{1}a_{2}-a_{3} \right)-{a_{1}}^{2}a_{4}>0$. Consequently, $E_{3}$ is locally stable if it exists.

*S2.5. Biomass,* $y$*, and* $z$ *in the steady state*

When a species invades an empty system and then a second species successfully invades the system occupied by the first species, the following conditions are satisfied:

$y^{*} < \hat{y}_{1} <y_{0}$, (S.10)

$z^{*}= \hat{z}_{2} <z_{0}$. (S.11)

The biomasses of the two species in the steady state are given as follows:

$x_{1}=\left\{ \begin{matrix} \frac{K_{y}+\hat{y}_{1}}{r_{1}\hat{y}_{1}}D_{y}\left( y_{0}-\hat{y}_{1} \right) at E_{1} \\ \frac{K_{y}+y^{*}}{r_{1}y^{*}}D_{y}\left( y_{0}-y^{*} \right) at E_{3} \end{matrix} \right.$ (S.12)

$x_{2}=\left\{ \begin{matrix} \frac{K_{z}+y^{*}}{r_{2}z^{*}}\left\{ D_{z}\left( z_{0}-\hat{z}_{2} \right) \right\} at E_{2} \\ \frac{K_{z}+y^{*}}{r_{2}z^{*}}\left\{ D_{z}\left( z_{0}-z^{*} \right) \right\}+D_{y}\left( y_{0}-y^{*} \right) at E_{3} \end{matrix} \right.$ (S.13)

Because of the conditions defined by Eqs. (S.10) and (S.11), the steady state biomass of a species is always higher in the presence of the other species than when it exists alone.

**S3.** **The invasion condition and the minimum value of** $\boldsymbol{-\Delta G}\boldsymbol{^{\circ}}$ **for successful invasion**

*S3.1. The invasion condition*

We explored two invasion conditions: (1) when a single species attempts to invade an empty system, and (2) when the species attempts to invade a system occupied by other species. The analysis was as follows:

[1] Consider when a positive but small number of organisms of Species 1 ($x_{1}=\epsilon$, where $\epsilon$ is a positive but small number) invade the steady state without species, $E_{0}$. Depending on the values of the parameters, Species 1 may increase in abundance (successful invasion) or decrease in number and become extinct (unsuccessful invasion).

[2] If Species 1 is able to invade the empty system, the system converges to $E_{1}$, at which only $x_{1}$ exists: $\left( \hat{x}_{1}, 0,\hat{y}_{1}, \hat{z}_{1} \right)$.

[3] Similarly, consider when a small number of organisms of Species 2 ($x_{2}=\epsilon$) invade the steady state without species, $E_{0}$: $\left( 0,0,y_{0},z_{0} \right)$. Depending on the parameter values, Species 2 may increase in abundance (successful invasion) or decrease in number and become extinct (unsuccessful invasion).

[4] If Species 2 is able to invade the empty system, the system converges to $E_{2}$, at which only $x_{2}$ exists: $\left( 0,\hat{x}_{2},\hat{y}_{2},\hat{z}_{2} \right)$.

[5] If a small number of organisms of Species 2 ($x_{2}=\epsilon$) invade a system in steady state $E_{1}$, then, depending on the parameter values, Species 2 may or may not successfully invade the system.

[6] If Species 2 is able to invade a system in steady state $E_{1}$, the system converges to $E_{3}$, in which both species coexist: $\left( x_{1}^{*},x_{2}^{*},y^{*},z^{*} \right).$

[7] Similarly, if a small number of organisms of Species 1 ($x_{1}=\epsilon$) invade a system in steady state $E_{2}$, then, depending on the parameter values, Species 1 may or may not successfully invade the system.

[8] If Species 1 is able to invade a system in steady state $E_{2}$, the system converges to $E_{3}$, in which both species coexist.

[9] If Species 1 is able to invade a system in steady state $E_{1}$ and Species 2 is able to invade a system in steady state $E_{2}$, the final steady state is the same for these two cases, namely that $E_{3}$: $\left( x_{1}^{*},x_{2}^{*},y^{*},z^{*} \right)$ is unique.

*S3.2. Minimum* $-\Delta G^{\circ}$ *for successful invasion*

Using a similar argument to that used for deriving Eqs. (5a) and (5b), it is possible to calculate the minimum $-\Delta G^{\circ}$ required for successful invasion when a single species attempts to invade an empty system, $\theta_{i,solo}$:

$\theta_{1,solo}=\frac{m_{1}}{c_{1}r_{1}}\left( 1+\frac{K_{y}}{y_{0}} \right)+RT\ln\left( \alpha_{1}\frac{{(z_{0})}^{n}}{y_{0}} \right)$, (S.14a)

$\theta_{2,solo}=\frac{m_{2}}{c_{2}r_{2}}\left( 1+\frac{K_{z}}{z_{0}} \right)+RT\ln\left( \alpha_{2}\frac{1}{z_{0}} \right)$ , (S.14b)

where $y_{0}$ and $z_{0}$ are the values of $y$ and $z$ at the empty steady state $E_{0}$: $\left( 0,0,y_{0},z_{0} \right)$. Eq. (S.14a) gives the boundary between the region in which steady state $E_{0}$ is stable and that in which steady state $E_{1}$ is stable (see point [2] in section S3.1, above). Similarly, Eq. (S.14b) gives the boundary between the regions in which steady states $E_{0}$ and $E_{2}$ are stable.

Consider the case in which a small number of cells of Species 1 invade a system in a steady state with Species 2 only, i.e., $E_{2}$: $\left( 0,\hat{x}_{2},\hat{y}_{2},\hat{z}_{2} \right)$. Species 1 can increase in number if $-\Delta G_{1}^{o}>\theta_{1, acc}$, whereas it will decrease if the opposite inequality holds. Similar to Eq. (S.14a),

$\theta_{1, acc}=\frac{m_{1}}{c_{1}r_{1}}\left( 1+\frac{K_{y}}{\hat{y}_{2}} \right)+RT\ln\left( \alpha_{1}\frac{\left( \hat{z}_{2} \right)^{n}}{\hat{y}_{2}} \right)$ , (S.15a)

which is derived from Eq. (4a) by setting the net energy acquisition per unit time within braces of $x_{1}$ to be zero. However, $\hat{y}_{2}$ and $\hat{z}_{2}$ in Eq. (C.2a) indicates the abundance of $y$ and $z$ in the steady state with Species 2 only. Since species 1 is absent, $\hat{y}_{2}=y_{0}=I_{y}/D_{y}$ holds. However, $\hat{z}_{2}$ is affected by the presence of species 2. From Eq. (4d), we can see that $\hat{z}_{2}$ $<z_{0}$, because of the negative contribution of the third term in the right-hand side.

Comparing Eq. (S.14a) and (S.15a), we can conclude that $\theta_{1, acc}<\theta_{1, solo}$. This implies that the presence of species 2 helps the invasion of species 1, or help species 1 from extinction.

Now, consider the case in which a small number of species 2 invade the steady state with species 1 only, i.e., $E_{1}$: $\left( \hat{x}_{1},0,\hat{y}_{1},\hat{z}_{1} \right)$. The minimum value of $-\Delta G_{2}^{o}$ required for successful invasion can be calculated from Eq. (4b) as follows:

$\theta_{2,acc}=\frac{m_{2}}{c_{2}r_{2}}\left( 1+\frac{K_{z}}{\hat{z}_{1}} \right)+RT\ln\left( \alpha_{2}\frac{1}{\hat{z}_{1}} \right)$ . (S.15b)

Here, $\hat{z}_{1}$ calculated from Eq. (5a) is the abundance in the steady state with Species 1 only. $\hat{z}_{1}>z_{0}$ because of the presence of the positive contribution of the second term on the right-hand side of Eq. (2d); here, the inequality is opposite to that in the previous case. Hence, $\theta_{2, acc}<\theta_{2, solo}$. Again, the mimimum $-\Delta G_{2}^{o}$ required for successful invasion is smaller in the presence of Species 1.

The first terms on the right-hand side of Eqs. (S.15a) and (S.15b) can be regarded as the effect without the ARP. Comparing these terms shows that, even without the ARP effect, $\theta_{2, acc}$ is smaller than $\theta_{2, solo}$. Taking account of the ARP effect, the second terms of Eqs. (S.15a) and (S.15b) increases the magnitude of the difference between $\theta_{2, acc}$ and $\theta_{2, solo}$. Importantly, $\theta_{1, acc}$ is smaller than $\theta_{1, solo}$ only when the ARP effect contributes to the energy acquisition per reaction.

**S4. The presence of one species benefits the second species**

Our analysis showed the following mutualistic behaviors between Species 1 and 2:

[1] With some parameter values, Species 1 cannot invade an empty system $E_{0}$, $\left( x_{1},x_{2} \right)=(0, 0)$. However, species 2 can invade $E_{0}$ and reach the steady state with Species 2 only, i.e, $E_{2}, \left( x_{1},x_{2} \right)=(0, \hat{x}_{2})$. Then, depending on the parameter values, Species 1 may be able to invade the system in state $E_{2}, \left( x_{1},x_{2} \right)=(0, \hat{x}_{2})$, increasing to reach the coexistence steady state, $E_{3}, \left( x_{1},x_{2} \right)=\left( {x_{1}}^{*},{x_{2}}^{*} \right)$. Here Species 1 only continues to exist when Species 2 is also present in the system. In this case, Species 1 benefits from the presence of Species 2.

[2] Similarly, for some parameter values, Species 2 cannot invade the empty system $E_{0}$ but Species 1 is able to invade the system, which converges to the steady state with Species 1 only, i.e., $E_{1}= \left( x_{1},x_{2} \right)=(\hat{x}_{1}, 0)$. Species 2 may then be able to invade the system in state $E_{1}$, depending on the parameter values, leading to the coexistence steady state $E_{3}$. In this case, Species 2 benefits from the presence of Species 1.

[3] The steady-state biomass of Species 1 is greater in the coexistence steady state $E_{3}$ than in $E_{1}$, the steady state with Species 1 only (i.e., $\hat{x}_{1}<$ $x_{1}^{*}$) (Supporting Information 2.5). In this case, Species 1 benefits from the presence of Species 2.

[4] Similarly, the steady state biomass of Species 2 is greater in the coexistence steady state $E_{3}$ than in $E_{2}$, the steady state with Species 2 only (i.e., $\hat{x}_{2}<x_{2}^{*}$) (Supporting Information 2.5). In this case, Species 2 benefits from the presence of Species 1.

We define the mutualism between the two species in our model as the combination of [1] and [4] and the combination of [2] and [3].

**S5. Parameter dependence of the invasion**

We numerically examined the parameter dependence of the ability of the species to invade an ecosystem. Using numerical simulations, we explored the steady state of Eq. (4) for 5000 parameter sets that were generated at random with a uniform distribution across the ranges listed in Table A1. The values of $q_{i}$ and $c_{i}$ were fixed for the following reasons: $q_{i}$ affects the dynamics of Eq. (4) but does not affect the stable steady state to which the system finally converges after a sufficiently long time; and the dependence of the ability to invade on $c_{i}$ should be the same as that for $r_{i}$. To highlight how the different parameter values influenced the effect of ARP on $y$ and $z$, we also set $\alpha_{i}$ to 1. In general, the increase in $\alpha_{1}$ and $\alpha_{2}$ negatively affects the growth of Species 1 and 2, respectively. For the 5000 parameter sets, the system eventually reached the steady state of type $E_{0}$ with 1742 sets, type $E_{1}$ with 1045 sets, type $E_{2}$ with 1479 sets, and type $E_{3}$ with 734 sets.

Apart from $T$ and $-\Delta G_{i}^{^{\circ}}$, each parameter was increased incrementally by a factor of 10 and the steady states were again explored to see whether the system allowed more species to occupy or exclude microbes. In addition, $T$ was incremented by 10 K and $-\Delta G_{i}^{^{\circ}}$ by 50 kJ mol^−1^. The code for Mathematica of this simulation is provided as Supplementary Source Code.

Fig. S1 summarizes the transitions in the steady states resulting from the increase in each parameter. The increases in $I_{y}$, $I_{z}$, $D_{z}$, $r_{1}$, $r_{2}$, $-\Delta G_{1}^{^{\circ}}$, $-\Delta G_{2}^{^{\circ}}$, and $n$ allowed one or two species to invade, with transitions from $E_{0}$ to the other states ($E_{1}$, $E_{2}$, or $E_{3}$) (Fig. S1(a)). The positive effects of the increase in $D_{z}$ and $n$ on the invasiveness of Species 1 were distinctive features of the growth under the influence of the ARP effect, because the fast removal of $z$ and increase in *n* enhanced the energy gain per reaction by increasing the ARP. The increase in *n* was also beneficial to Species 2, which was able to utilize more $z$, leading to a transition from $E_{0}$ to $E_{3}$.

The increases in $I_{y}$, $r_{2}$, $-\Delta G_{2}^{^{\circ}}$, and $n$ resulted in coexistence of the species (a transition from $E_{1}$ to $E_{3}$) (Fig. S1 (b)), whereas increases in $D_{y}$, $K_{y}$, and $m_{1}$ resulted in the extinction of Species 1 (a transition from $E_{1}$ to $E_{0}$). Interestingly, the increase in $I_{z}$ resulted in either coexistence of the two species or the extinction of Species 1 because it can negatively affect the ARP of Species 1 due to the accumulation of $z$ and positively affect the growth of Species 2 through the increased supply of resource.

The increases in $I_{y}$, $r_{1}$, $r_{2}$, $-\Delta G_{1}^{^{\circ}}$, $-\Delta G_{2}^{^{\circ}}$, and $n$ resulted in the coexistence state (the transition from $E_{2}$ to $E_{3}$) (Fig. S1 (c)), indicating that the enhanced growth of Species 2 could increase its realized niche (as shown in Fig. 5). The increases in $D_{z}$, $K_{z}$, and $m_{2}$ resulted in the extinction of Species 2 (a transition from $E_{2}$ to $E_{0}$).

The increases in $D_{y}$, $D_{z}$, $K_{y}$, $K_{z}$, $m_{1}$, or $m_{2}$ resulted in the exclusion of one or both species (Fig. S1 (d)). A change in any one of these parameters directly reduced the growth of one of the species; this then caused the niche of the other species to shrink, eventually resulting in the extinction of both species.

Overall, the increase in *D_y_* and *D_z_* negatively influences the existence and local stability of *E*_3_. An increase in *D_y_* leads to the rapid removal of *y*, resulting in the limitation of the growth of Species 1 and, in turn, Species 2. An increase in *D_z_* positively works on the existence of Species 1 due to the removal of its by-product, but negatively influences the existence of Species 2.

**S6. Speed of the catabolic reactions in the steady state**

In our model, the speed of the catabolic reactions of Species1 and 2 are given by the Michaelis–Menten formulas for the microbes’ enzymes. In the steady state given by Eq. (4), the reaction rate must be equal to the rate of exchange with the environment external to the system, as follows:

$\frac{r_{1}y}{K_{y}+y}x_{1}=\left\{ \begin{matrix} D_{y}\left( y_{0}-\hat{y}_{1} \right) at E_{1} \\ D_{y}\left( y_{0}-y^{*} \right) at E_{3} \end{matrix} \right.$ , (S.16a)

$\frac{r_{2}z}{K_{z}+z}x_{2}=\left\{ \begin{matrix} D_{z}\left( z_{0}-\hat{z}_{2} \right) at E_{2} \\ D_{z}\left( z_{0}-z^{*} \right)+D_{y}\left( y_{0}-y^{*} \right) at E_{3} \end{matrix} \right.$. (S.16b)

As Eqs. (S.12) and (S.13) hold, the invasion by a second species that removes a by-product released from the energy-harvesting reaction of a species already present can increase the reaction speed for $y$. When a species is alone in the system, the ARP effect can have a positive or negative influence on the steady state concentrations of $\hat{y}_{1}$ or $\hat{z}_{2}$, depending on the parameter values; thus, the ARP effect does not always intensify the flux or resources utilized for the production of energy. The mutualistic interaction between species that emerges from the ARP effect can increase the flux of resources utilized by the species to harness energy, potentially contributing to the development of a functioning ecosystem.
