# Supplementary material for: The fitness of chemotrophs increases when their catabolic by‐products are consumed by other species
Source: Ecol Lett. 2019 Oct 14;22(12):1994–2005. doi: 10.1111/ele.13397 (PMC6899997; doi:10.1111/ele.13397)
Supplement: Supplementary file 5 [file ELE-22-1994-s005.docx]

**Supplemental Figure Captions**

**Fig. S1.** Changes in the steady state caused by increases in the parameters (*T* by 10 K, −Δ*G_i_*º by 50 kJ mol^−1^, and the other parameters by a factor of ten). The initial steady states were (a) *E*_0_, (b) *E*_1_, (c) *E*_2_, and (d) *E*_3_.

**Fig. S2** The steady-state biomass and the increase in the biomass resulting from the presence of the mutualistic partner. The horizontal axis represents *r*_2_, the maximum catalytic rate for Species 2. Inflows *I_y_* and *I_z_* are fixed at 10^-4^ and 1.2 × 10^-8^ mmol L^-1^ h^-1^, respectively. (Upper panel) The invasion of an empty system. (Middle panel) The invasion of a system in the presence of the mutualistic partner species. (Lower panel) The increased steady-state biomass resulting from the presence of the mutualistic partner. The steady-state biomasses were calculated with the other parameters fixed at the standard values listed in Table S1.
